# A Comprehensive Phytochemical Analysis of Terpenes, Polyphenols and Cannabinoids, and Micromorphological Characterization of 9 Commercial Varieties of *Cannabis sativa* L.

**DOI:** 10.3390/plants11070891

**Published:** 2022-03-27

**Authors:** Eugenia Mazzara, Jacopo Torresi, Gelsomina Fico, Alessio Papini, Nicola Kulbaka, Stefano Dall’Acqua, Stefania Sut, Stefania Garzoli, Ahmed M. Mustafa, Loredana Cappellacci, Dennis Fiorini, Filippo Maggi, Claudia Giuliani, Riccardo Petrelli

**Affiliations:** 1 Chemistry Interdisciplinary Project (CHIP), School of Pharmacy, University of Camerino, Via Madonna delle Carceri, 62032 Camerino, Italy; eugenia.mazzara@unicam.it (E.M.); jacopo.torresi@unicam.it (J.T.); ahmed.mustafa@unicam.it (A.M.M.); loredana.cappellacci@unicam.it (L.C.); riccardo.petrelli@unicam.it (R.P.); 2 Department of Pharmaceutical Sciences, University of Milan, Via Mangiagalli 25, 20133 Milan, Italy; gelsomina.fico@unimi.it (G.F.); claudia.giuliani@unimi.it (C.G.); 3 Ghirardi Botanic Garden, Department of Pharmaceutical Sciences, University of Milan, Via Religione 25, 25088 Toscolano Maderno, Italy; 4 Department of Biology, University of Florence, Via La Pira 4, 50121 Florence, Italy; alessio.papini@unifi.it; 5 Società Agricola Everweed Di G.Di Vietri & C. SS, Frazione Conti 2, 63857 Amandola, Italy; everweed.nicola@gmail.com; 6 Natural Product Laboratory, Department of Pharmaceutical and Pharmacological Sciences, University of Padova, 35131 Padova, Italy; stefano.dallacqua@unipd.it (S.D.); stefania.sut@unipd.it (S.S.); 7 Department of Drug Chemistry and Technology, Sapienza University, 00185 Rome, Italy; stefania.garzoli@uniroma1.it; 8 Department of Pharmacognosy, Faculty of Pharmacy, Zagazig University, Zagazig 44519, Egypt; 9 Chemistry Interdisciplinary Project (CHIP), School of Science and Technology, University of Camerino, Via Madonna delle Carceri, 62032 Camerino, Italy; dennis.fiorini@unicam.it

**Keywords:** *Cannabis sativa* L., chemovars, secondary metabolites, trichomes, residual by-products

## Abstract

New hemp (*Cannabis sativa* L.) strains developed by crossbreeding selected varieties represent a novel research topic worthy of attention and investigation. This study focused on the phytochemical characterization of nine hemp commercial cultivars. Hydrodistillation was performed in order to collect the essential oils (EO), and also the residual water and deterpenated biomass. The volatile fraction was analyzed by GC-FID, GC-MS, and SPME-GC-MS, revealing three main chemotypes. The polyphenolic profile was studied in the residual water and deterpenated biomass by spectrophotometric assays, and HPLC-DAD-MS^n^ and ^1^H-NMR analyses. The latter were employed for quali–quantitative determination of cannabinoids in the deterpenated material in comparison with the one not subjected to hydrodistillation. In addition, the glandular and non-glandular indumentum of the nine commercial varieties was studied by means of light microscopy and scanning electron microscopy in the attempt to find a possible correlation with the phytochemical and morphological traits. The EO and residual water were found to be rich in monoterpene and sesquiterpene hydrocarbons, and flavonol glycosides, respectively, while the deterpenated material was found to be a source of neutral cannabinoids. The micromorphological survey allowed us to partly associate the phytochemistry of these varieties with the hair morphotypes. This research sheds light on the valorization of different products from the hydrodistillation of hemp varieties, namely, essential oil, residual water, and deterpenated biomass, which proved to be worthy of exploitation in industrial and health applications.

## 1. Introduction

Hemp (*Cannabis sativa* L.) is a versatile crop, for a long time cultivated in different parts of the world, and its cultivation has recently been considered as a good opportunity for agriculture production. Legally cultivated hemp has been selected in order to keep the psychotropic δ-9-tetrahydrocannabinol (THC) at levels lower than 0.2% [1]. The main non-psychotropic cannabinoid found in hemp, namely, cannabidiol (CBD), has recently been included in the list of the ingredients that can be used in cosmetics, thus offering new opportunities for the development of hemp products. In fact, up to now, the use of CBD has been approved in the cosmeceutical industry [2]. For medicinal applications, CBD has been registered as a drug (Epidiolex^®^) in the EU for the treatment of some epilepsy forms [3]. CBD regulation in the food or food supplements area is not uniform in European countries; thus, CBD products can be produced in some countries as food supplements, but they cannot be in others. Many studies have been developed in the last few years on CBD’s potential uses as medicine, but CBD-fortified or CBD-labeled health products and CBD-associated health claims lack a rigorous scientific foundation [4]. At present, many research groups are studying CBD and other cannabinoids for possible applications in healthcare, medicine, food supplements, and cosmetics. In this regard, improving the knowledge on different hemp varieties or cultivars to obtain extracts enriched in specific phytoconstituents may be advantageous.

Moreover, hemp inflorescences, usually regarded as waste material of the fiber industry, are instead valuable sources of volatile constituents, secreted in the form of essential oils (EO), that can be obtained by distillation procedures [5]. Specifically, hemp is endowed with secretory structures, namely trichomes, representing the main site to produce secondary metabolites. Among them, cannabinoids and terpenes are contained in a sort of resin released by capitate-stalked glandular hairs localized on flower bracts and, to a minor extent, by capitate sessile and bulbous trichomes occurring also on other vegetative organs [6]. Notably, volatile terpenes of hemp glandular trichomes are recovered as a yellowish and odorous EO [7,8]. Morphological descriptions of hemp trichomes play a significant role in forensic investigations and legal/illegal cannabis identification. Given the high variability in *C. sativa* morphological features, the study of such anatomical characteristics appears to be necessary and useful for varieties classification and characterization [9]. For these reasons, traditional optical, electron, and fluorescence microscopy, and the more common Raman spectroscopy [10], have been employed for the localization and analysis of cannabinoids in cannabis trichomes.

As a source of certified new products, hemp EO is gaining more interest from agrochemical, cosmeceutical, pharmaceutical, and nutraceutical perspectives [11]. Indeed, this EO exhibited repellent, acaricidal [12,13], and insecticidal activity [14,15], to be exploited in the development of safe botanical pesticides in organic farming and parasite-control programs, as a promising alternative to conventional synthetic agents [16]. Hemp EO can also be employed as a scent in cosmetics, such as soaps and perfumes [17]. Moreover, the displayed antifungal, anti-inflammatory [18], antiprotozoal and antioxidant [19] effects support its use in infectious diseases, in the form of dermatological preparations and as an ingredient in protective masks against COVID-19 [18]. In addition, recent studies on the use of plant EOs as natural food preservatives evidenced the capability of hemp EO to enhance the shelf-life of trout fillets, due to its antimicrobial properties, which were improved by nano-encapsulation [20]. These results support further applications of hemp EO in green active packaging to maintain food safety and quality.

Hemp inflorescence distillation of EO produces two by-products, namely, residual water and deterpenated plant material, which are currently under-studied. The aqueous residues could be rich in phenolic compounds and can be extracted with safe solvents, such as ethanol and water [21]. Hemp polyphenols are interesting biomolecules, able to reduce the progression of cardiovascular and neurodegenerative diseases, asthma, inflammatory conditions, tumors, and others [22]. Among cannabis-typical flavones, cannflavin A and B are particularly attractive compounds, with anti-inflammatory [23], antiparasitic [24], anticancer, and antiviral properties [25]. Flavonoids contribute to plant protection, especially from UV light, due to their antioxidant and radical scavenger activity. In fact, cannflavin A was detected at a high level in hemp varieties under the influence of strong solar radiation and cold temperatures [26].

The inflorescences and, to a minor extent, the leaves can also be considered as good sources of cannabinoids, especially CBD. In hemp plant material, cannabinoids are mostly present in the acidic form, and the heating of biomass at temperatures near 100 °C induces the decarboxylation of these compounds. So, this material can be used after distillation for the extraction of CBD and other minor cannabinoids. Indeed, some authors recently affirmed that hemp deterpenated biomass should no longer be considered as a waste [27], since it represents a source of phytocannabinoids [28]. CBD is nowadays of great interest for several applications, and less investigated minor cannabinoids, such as cannabigerol (CBG), can be equally promising on a pharmaceutical level. Specific chemovars characterized by other cannabinoids represent a challenging research area and can be the source of new appealing products [29]. For example, the French cultivar Santhica, characterized by a significant CBG content, could be employed to develop new hemp strains as reservoirs of this cannabinoid [30]. Actually, CBD and CBG, used in combination, showed antioxidant and anti-inflammatory activity [31], as well as antidiabetic and antimicrobial potential [32,33]. In particular, CBG reduced cachexia caused by chemotherapy [34], and was found to be active against several tumoral cell lines, including glioblastoma [35]. In addition, CBG could be a candidate in therapy against inflammatory bowel disease [36].

Most of the published papers on *C. sativa* are related to certified industrial hemp varieties or legal cannabis for recreational and therapeutic purposes, while studies on commercial cultivars of cannabis with low THC content are scarce. The crossbreeding of selective varieties with interesting terpenoids and phytocannabinoids profiles could allow obtaining new hemp breeding lines with diverse medicinal and pharmacological properties, and with potential industrial applications, such as antiseptics and biopesticides [37]. On this basis, this work aims to carry out a complete phytochemical characterization of several commercial hemp strains, cultivated for research purposes and to produce craft beers. Our study explored the opportunity to obtain three high-value products from hydrodistillation of the hemp inflorescences, namely, EO, residual water, and deterpenated plant material. The EO and residual water are sources of volatile terpenes and polyphenols, respectively. Moreover, the strong heating during distillation may induce the decarboxylation of cannabinoids in the plant deterpenated material. In this study, nine different hemp commercial varieties have been used as training model plants to establish the value of this complete extraction process. This research also aimed at offering new opportunities to develop a smart extraction approach to valorize hemp by-products, supporting their further utilization in the pharmaceutical, nutraceutical, cosmeceutical, and food sectors.

Furthermore, to substantiate this approach, a comprehensive micromorphological and histochemical study of glandular and non-glandular hairs has been carried out in the attempt to correlate the phytochemistry and morphological traits of these commercial varieties with the morphotype, abundance, distribution, and secretory products of trichomes.

## 2. Results and Discussion

### 2.1. Analysis of the Volatile Fraction

#### 2.1.1. Analysis of EOs Yields

The yields of the EOs from the nine studied commercial varieties ranged from 0.485% *w*/*w* in Pablito to 1.814% *w*/*w* in Amnesia Cookies (Table 1). It is worth noting that in the literature, the maximum yield value for hemp EO was 0.60% [38], so the values registered in this work were considerably high, with respect to those obtained for the EOs from the certified European hemp varieties [7,39]. The EO yield can be influenced by several factors, such as genetics, plant biomass status, environmental and climatic conditions, post-harvesting, drying, etc. For this work, only non-pollinated female inflorescences were processed, and this aspect could explain the significant EO yields obtained. In fact, it is known to hemp growers, and it has also been demonstrated, that the pollination of *Cannabis* plants should be prevented to ensure EO yields twice those deriving from pollinated flowers [40]. Some stress conditions, such as the temperature changes involved in the harvesting periods of the nine varieties, could enhance the secretion of EO, with cannabinoids and terpenes produced as plant defense agents [41]. Moreover, slow air drying in a dark environment prevented humidity and degradation of the biomass by microorganisms, by protecting the inflorescences against sun light, with the consequent preservation of the aroma and organoleptic features of each variety.

#### 2.1.2. GC-FID Quantitative Determination of Hemp EO Main Constituents

The quantitative GC-FID analysis results for the nine EOs, shown in Table 1, highlight the presence of α-pinene, myrcene, terpinolene, and (*E*)-caryophyllene as the major terpenes. The highest content of α-pinene was detected in Venom OG (21.2 g/100 g), while Amnesia Cookies proved to be the richest in myrcene (29.2 g/100 g). The best concentration value of terpinolene was found in Lemon Conti Kush New (30.5 g/100 g), while (*E*)-caryophyllene reached the greatest amount in Fresh Mountain (19.7 g/100 g). The limonene, α-humulene, and caryophyllene oxide percentages were lower than those of the above compounds (for a total of 9.2 g/100 g in White Shark, 8.6 g/100 g in Amnesia Cookies and 8.0 g/100 g in 24 K). The other quantified terpenes, namely, 1,8-cineole and (*E*)-β-ocimene, were detected at a very low level in all the investigated EOs. CBD content ranged from 2.3 g/100 g in Amnesia Cookies to 5.5 g/100 g in White Shark EOs. It is worth noting that in all the analyzed EOs, the THC content was within the limit of 0.2%, which is far below that stated by EU regulation for industrial hemp biomass (Table 1). 

#### 2.1.3. GC-MS Analysis of EOs

A comprehensive GC-MS analysis was carried out using two columns of different polarities, namely, HP-5MS and DB-WAX, to obtain an overview of the chemical profiles of the nine EOs. The results of the two analyses were comparable, confirming the presence of the same main compounds in each variety. Interestingly, the more apolar HP-5MS stationary phase allowed us to obtain, for several components, higher relative abundances, with respect to those found by employing the more polar DB-WAX column. For this reason, Table 2 reports the percentage values provided by the analysis performed with the HP-5MS column.

Among the monoterpenes, α-pinene, myrcene, and terpinolene were again the most abundant compounds detected in the EOs from the nine commercial varieties. Only one of them, namely Venom OG, showed prevalence of α-pinene over the other terpenes, as reported in the GC-FID analysis (Section 2.1.2). Indeed, this component accounted for 23.3% of the whole chemical profile, followed by (*E*)-caryophyllene (17.1%). Myrcene was the predominant constituent of the EOs from Amnesia Cookies, Fresh Mountain, White Shark, and Lemon Conti Kush (27.1%, 26.0%, 21.5%, and 14.9% of the total composition, respectively) confirming the GC-FID outcomes (Table 1). Along with myrcene, Amnesia Cookies’ EO was characterized by a significant content of α-pinene (24.6%) and (*E*)-caryophyllene (15.6%). In addition to myrcene, other abundant constituents were (*E*)-caryophyllene (16.0%) and α-pinene (11.0%) in Fresh Mountain, and terpinolene (11.8%), (*E*)-caryophyllene (11.4%), α-pinene (11.3%) and limonene (10.2%) in White Shark. Myrcene was followed by α-pinene (12.5%) and (*E*)-caryophyllene (10.7%) in Lemon Conti Kush. Differently from the latter, the Lemon Conti Kush New EO presented terpinolene as the predominant component, accounting for a remarkable 30.2% of the total composition, followed by myrcene (9.5%). This EO was also the one with the highest content of terpinolene among all the EOs, as shown by the GC-FID analysis results.

Unlike the previous ones, 24 K, Gorilla Glue, and Pablito EOs were dominated by sesquiterpenes. Among them, (*E*)-caryophyllene was the most prevalent compound, in line with GC-FID analysis, representing 19.9%, 17.6%, and 15.5% of EO, respectively. It was followed by 10-*epi*-γ-eudesmol (10.2%) and guaiol (9.7%) in 24 K, and by selina-3,7(11)-diene (12.5%) and selina-4(15),7(11)-diene (7.1%) in Gorilla Glue. With respect to the other EOs, Pablito contained a higher relative percentage of α-bisabolol (8.6%).

Regarding cannabinoids, the only compounds detected in the nine commercial varieties’ EOs were CBD and cannabichromene (CBC). While CBC was found only in traces in all the EOs, CBD content ranged from 0.1% to 0.6%, except in Venom OG, where it reached 1.0% (Table 2).

The above-described GC-MS outcomes highlight a certain variability in the chemical profiles of the investigated EOs, among each other and also, in some cases, with respect to those obtained by the certified industrial hemp varieties which were the relatives of the nine commercial cultivars considered in this study. In fact, the Pablito EO was characterized by the prevalence of (*E*)-caryophyllene and other sesquiterpenes, while its original monoecious variety Santhica 70 was reported to be richer in monoterpenes, especially myrcene, although its EO was obtained by steam distillation [42]. 24 K and Gorilla Glue, deriving from Carmagnola CS, presented the sesquiterpenes class and, particularly, (*E*)-caryophyllene as predominant constituents; notably, Carmagnola EO showed a higher amount of monoterpenes (especially myrcene) in the case of steam-distilled fresh inflorescences [43], but a higher sesquiterpene content (notably (*E*)-caryophyllene) when hydrodistillation of dry inflorescences was carried out [44]. Regarding Lemon Conti Kush, Fresh Mountain, and Amnesia Cookies, originated by the Kompolti variety, a considerable abundance of myrcene and other monoterpenes was detected in their EOs. This was in accordance with some literature findings in which the Kompolti EO from dried inflorescences appeared to be richer in the same compounds, rather than in sesquiterpenes [45]. So, it could be deduced that such differences in the distribution of mono- and sesquiterpenes in hemp EOs seem to be dependent on the extraction method and plant material status.

**Figure 1:**
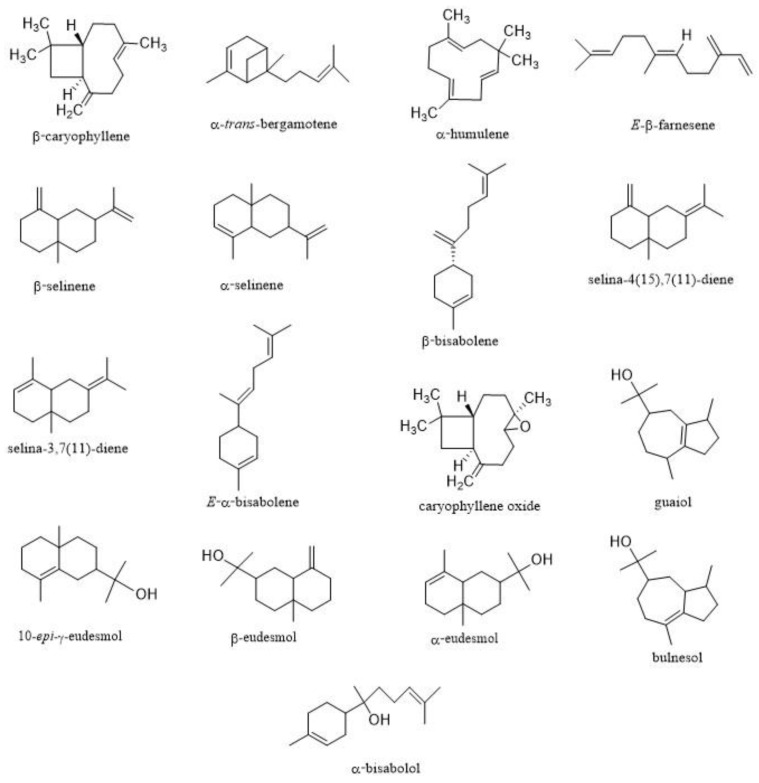
Chemical structures of the main sesquiterpenes found in the 9 EOs.

Interestingly, in some EOs from the nine commercial varieties, the presence of several sesquiterpenes structurally different from (*E*)-caryophyllene was found at noteworthy levels (Figure 1). Among them, 10-*epi*-γ-eudesmol and guaiol in 24 K, selina-3,7(11)-diene and selina-4(15),7(11)-diene in Gorilla Glue, and α-bisabolol in Pablito could be mentioned as the most representative compounds (Table 2). These sesquiterpenes were not frequent in hemp EOs from other studies. For instance, they were missing in the Kompolti EO analyzed by Novak et al. [46], while in Bertoli et al. [5], selina-3,7(11)-diene and α-bisabolol were detected, but in very low amounts (<1%), as for all the other minor sesquiterpenes identified in Carmagnola EO.

#### 2.1.4. Chiral GC-MS Analysis of EOs

The EOs from the nine commercial varieties were subjected to chiral GC-MS analysis to determine the enantiomeric distribution of α-pinene, β-pinene, limonene, linalool, (*E*)-caryophyllene and caryophyllene oxide. This analysis could be helpful to detect the origin of the EO samples based on the enantiomeric ratio shown by the main constituents. In this regard, Table 3 reports the ratios within the enantiomeric pairs of these chiral terpenes. Both the enantiomers of α-pinene and limonene were detected in all the varieties. For α-pinene, the predominance of the (+)-form was observed, while the (−)-enantiomeric form was the prevalent one in the case of limonene. No significant differences in the enantiomeric ratios were obtained for these two compounds among the nine studied varieties. On the other hand, β-pinene and linalool were characterized by higher variability in the enantiomeric ratios. In fact, the (+)-enantiomer of β-pinene was generally predominant, although the presence of only the (−)-form was found in EOs from two varieties, namely, 24 K and Gorilla Glue. Moreover, the prevalence of the (+)-enantiomer of linalool was detected, and it was the only recognized form in Fresh Mountain, Gorilla Glue and Venom OG EOs. It is worth noting that the elution order of the enantiomeric forms of α-pinene and β-pinene was compliant with that found in one of our previous works, where the same column was employed [8]. Interestingly, (+)-α-pinene and (+)-β-pinene, as the main enantiomers in the nine EOs, were reported to be endowed with antibacterial and antifungal properties [47]. Concerning sesquiterpenes, (*E*)-caryophyllene and caryophyllene oxide were present exclusively in the (−)-enantiomeric form (Table 3), as confirmed by another study on hemp EOs [8]. From the findings provided, it is possible to conclude that the enantiomeric ratio of the main volatile terpenes does not allow us to differentiate most of the varieties analyzed, apart from 24 K and Gorilla Glue, which showed the predominance of the (−)-β-pinene.

#### 2.1.5. SPME-GC-MS Analysis of Untreated Material

The dry female inflorescences (untreated material, UM) of the investigated hemp commercial varieties, provided by Everweed farm (Section 3.2), were evaluated for their organoleptic and aromatic features through the solid-phase microextraction (SPME) technique coupled to GC-MS. The detected volatile compounds belonged to the monoterpene and sesquiterpene classes, and, among them, α-pinene, myrcene, terpinolene, and (*E*)-caryophyllene represented the main fractions (Table 4). Myrcene dominated the volatile fractions of Pablito (35.2%), White Shark (44.6%), Fresh Mountain (39.2%), and Amnesia Cookies (37.2%) varieties, supporting the results on EOs (Section 2.1.2 and Section 2.1.3). Similarly, terpinolene was the most abundant compound in Lemon Conti Kush New (24.1%), and, to a minor extent, in White Shark (17.1%), as reported by the previous analyses on EOs. Concerning sesquiterpenes, 24 K, Gorilla Glue, Venom OG, and Lemon Conti Kush were characterized by higher levels of (*E*)-caryophyllene (50.3, 43.8, 36.5 and 25.7%, respectively). In compliance with the EOs’ GC-MS results, selina-3,7(11)-diene and selina-4(15),7(11)-diene were detected especially in Gorilla Glue (10.0 and 6.3%, respectively) and, in minor amounts, in Lemon Conti Kush and Lemon Conti Kush New (Table 4).

Regarding sesquiterpenes, the literature data suggest that a mountain environment could promote the production of these compounds, in particular (*E*)-caryophyllene and α-humulene, and other minor ones, such as selina-3,7(11)-diene [48]. In the cited study, dry inflorescences obtained from Kompolti grown in a mountainous area, analyzed with DVB/CAR/PDMS fiber as in the current work, presented significant amounts of the above sesquiterpenes, with respect to those obtained by lowland plants. In contrast to our research outcomes, the almost total absence of less common components, such as selina diene-, bisabolene-, and eudesmol-type sesquiterpenes, should be pointed out in other publications regarding the SPME analysis of inflorescences from several hemp varieties, including Carmagnola and Carmagnola CS [5]. Incidentally, the employed DVB/CAR/PDMS fiber was selected as the best one for the extraction of volatiles from *C. sativa* inflorescences [49].

### 2.2. Spectrophotometric Analysis of Lyophilized Extract and Deterpenated Material

The two hydrodistillation by-products, namely the water and plant biomass remaining in the round flask, were recovered separately and treated to obtain the lyophilized extracts (LE) and the dried deterpenated material (DM)**,** as reported in Section 3.3 and Section 3.5.1, respectively. LE and DM, obtained from the nine commercial varieties, were analyzed for their polyphenols and flavonoids content, and also for their antioxidant activity, in order to evaluate their possible reuse in pharmaceutical, nutraceutical, and cosmeceutical applications. In this regard, Table 5 shows the results of the spectrophotometric assays performed on the LE. The lowest and highest polyphenols and flavonoids contents were recorded in Fresh Mountain and Lemon Conti Kush New, respectively (between 40.4 and 72.2 mg of gallic acid equivalents (GAE) per g of dry extract (DE) for TPC, and from 18.2 to 46.3 mg of rutin equivalents (RE)/g DE for TFC). Moreover, the DPPH radical scavenging activity of LE varied from 90.3 to 143.0 mg of trolox equivalents (TE)/g DE in Fresh Mountain and Venom OG, respectively. As regards the studies on the antioxidant capacity of EU-approved hemp water extracts, Orlando et al. [50] found TPC, TFC, and DPPH values of 21.2 mg GAE/g_extract_, 7.1 mg RE/g_extract_ and 14.9 mg TE/g_extract_, respectively, for Futura 75 freeze-dried aqueous extract. As another example, a TPC value of around 55.0 mg GAE/g_extract_ was detected by Gunjevic et al. [51] in the lyophilized extract obtained from the residual water after Monoica hemp EO distillation. Notably, the TPC, TFC, and DPPH levels of all the nine varieties of the present study were much higher than those found by Orlando et al. [50]. The TPC values for only Fresh Mountain and Lemon Conti Kush LE were lower than those found by Gunjevic et al. [51], as well. Interestingly, the TPC and TFC levels for the LE obtained from all the investigated hemp varieties (apart from Fresh Mountain) were significantly above those ascribed to aqueous extracts of mulberry fruits (39.6 and 18.5 mg GAE/g_extract_ for TPC and TFC, respectively), which are well recognized and valorized for their antioxidant capacity [52].

As expected, due to the high-water solubility of these compounds, the polyphenols and flavonoids levels and the antioxidant activity in DM (Table 6) were lower than those belonging to LE. Nevertheless, DM could still represent potential sources of phenolic constituents, responsible for preventing the damaging effects of oxidation. The TPC values were in the range of 15.5–23.9 mg GAE per g of dry hemp (DW), reaching the minimum and maximum in Lemon Conti Kush and Venom OG DM, respectively. In the case of TFC, the results were between 8.3 mg RE/g DW in Amnesia Cookies and 22.0 mg RE/g DW in 24 K, while DPPH scavenging activity varied from 24.1 to 41.7 mg TE/g DW in Fresh Mountain and Venom OG, respectively. For comparative purposes, the same spectrophotometric tests were also performed on the dry inflorescences of Carmagnola CS, Santhica 70, and Kompolti, which were extracted with an ethanol-water 1:1 solution. As a result, the nine commercial varieties showed an improved TPC, TFC, and DPPH profile compared to the values obtained for the original certified ones (Table 6). These findings were confirmed by literature data. For instance, André et al. [53] indicated a TPC between 4.7 and 16.5 mg GAE/g DW for methanol:water extracts from Santhica 70 dried inflorescences. Moreover, Drinić et al. [54] reported TPC values ranging from 9.3 to 17.1 mg GAE/g DW for Helena aerial part extracts (50% ethanol in water). This solvent:water ratio, which was also chosen in the current research to carry out the tests, guaranteed the best extraction efficiency for phenolic constituents [54]. Based on the provided outcomes, the LE and DM from the nine hemp commercial varieties could represent interesting products that could be exploited for their antioxidant properties. Interestingly, LEs, currently recognized as waste obtained during distillation, represent valuable products to be used on an industrial level.

### 2.3. ^1^H-NMR Analysis of UM and DM

**Figure 2:**
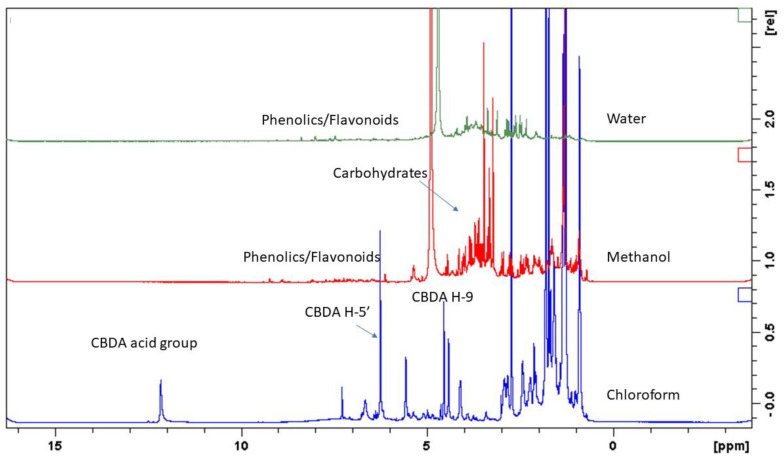
^1^H-NMR spectra showing the composition of UM chloroform, methanol and water extracts.

**Figure 3:**
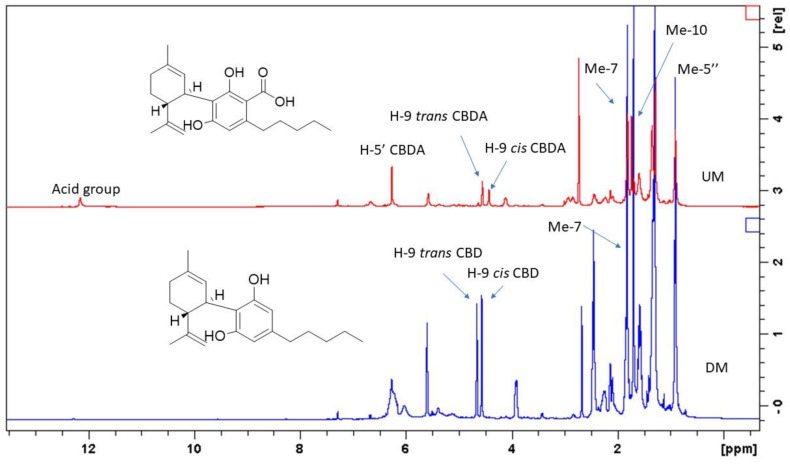
^1^H-NMR spectra showing the cannabinoids profiles of UM and DM chloroform extract.

To assess the multiple ways to extract valuable compounds from residual biomass resulting from distillation, the ^1^H-NMR approach was used as a first general attempt to establish what changes in composition were observable before and after the hydrodistillation of the nine hemp varieties. For this reason, UM and DM were used to perform ^1^H-NMR measurements, and samples were prepared using extractive solvents in sequence with increasing polarity. As a result, the UM chloroform extract showed the presence of cannabinoid acidic forms, the UM methanol extract presented a limited amount of phenolics and sugars, while the UM water extract was characterized by the presence of sugars (Figure 2). On the other hand, the DM chloroform extract showed the presence of cannabinoids, but the CBDA was in large part decarboxylated, being converted into CBD (Figure 3). The DM methanol and water extracts presented a limited amount of phenolics. Thus, the preliminary investigation by ^1^H-NMR revealed that DM, in comparison to UM, contained decarboxylated cannabinoids, with potential usefulness as a starting material for the extraction of these bioactive phytoconstituents.

### 2.4. HPLC-DAD-MS^n^ Analysis of LE, DM and UM

The HPLC-DAD-MS^n^ analysis of flavones in the nine commercial cultivars allowed us to identify 11 compounds, such as luteolin, apigenin, quercetin glycosides, and the hemp-specific flavonoids cannflavin A and B. Furthermore, the lignanamides cannabisin A and B were also identified and quantified. These compounds have been previously identified mainly in the seeds and are considered markers for hemp [55]. UM presented large variations in the amounts of these 11 phenolic derivatives. Among them, luteolin glucuronide was observed in a larger amount (more than 7.0 mg/g in Pablito UM and 4.2 mg/g in Lemon Conti Kush UM). Considering the sum of all the phenolic and lignanamide derivatives, Pablito UM presented these compounds in larger amounts, accounting for a total of more than 12.0 mg/g. Considering DM, a general decrease in the contents of the 11 detected compounds was observed, probably due to the washing away caused by water during the hydrodistillation process. Nevertheless, DM still contained a significant amount of certain phenolics. Notably, not all the compounds were washed away during EO distillation; these include cannflavins, which present low water solubility, and were quantified in a comparable amount in UM and DM of most varieties. On the other hand, glycosidic flavonoids, such as luteolin glycosides or rutin, which are in general more soluble in water at high temperatures, were less present in DM than in UM (Table 7).

LE presented significant amounts of phenolic compounds (Table 8). Very high levels of rutin have been observed in Venom OG, White Shark, 24 K, and Lemon Conti Kush LE. Other varieties, for example Fresh Mountain, contained only a limited concentration (0.3 mg/g) of rutin. Vitexin-2″-O-glucoside was observed in significant amounts in Venom OG (5.0 mg/g), Lemon Conti Kush (3.5 mg/g), and 24 K (3.2 mg/g). As expected, cannabinoids were present in negligible amounts in LE, due to their poor water solubility.

UM and DM were also studied for their cannabinoid composition, and the results are summarized in Table 9. As previously indicated by the preliminary investigation by ^1^H-NMR all the varieties were rich in acidic forms of cannabinoids, mostly CBDA. The cultivar Pablito presented CBGA as the most abundant cannabinoid. Very limited amounts of δ-9-THC have been detected, and the maximum observed levels were all below 0.2 mg/g. Comparing the levels of cannabinoids in UM and DM, most of the CBDA was converted to CBD in DM. In fact, DM possessed a larger amount of CBD, compared to the corresponding UM, due to decarboxylation reaction and conversion of CBDA in CBD. The cannabinoid levels were remarkably higher with respect to some data published on Finola and Futura varieties [56] and were comparable with those of other studies reporting on the amount of CBD in several hemp varieties cultivated in Slovenia [57].

### 2.5. Multivariate Statistical Analysis of EOs and UM

PCA was carried out to better visualize, within the nine commercial varieties, the behaviors of the main constituents belonging to the three studied fractions, namely, terpenes, polyphenols, and cannabinoids, in the plant biomass (inflorescences).

**Figure 4:**
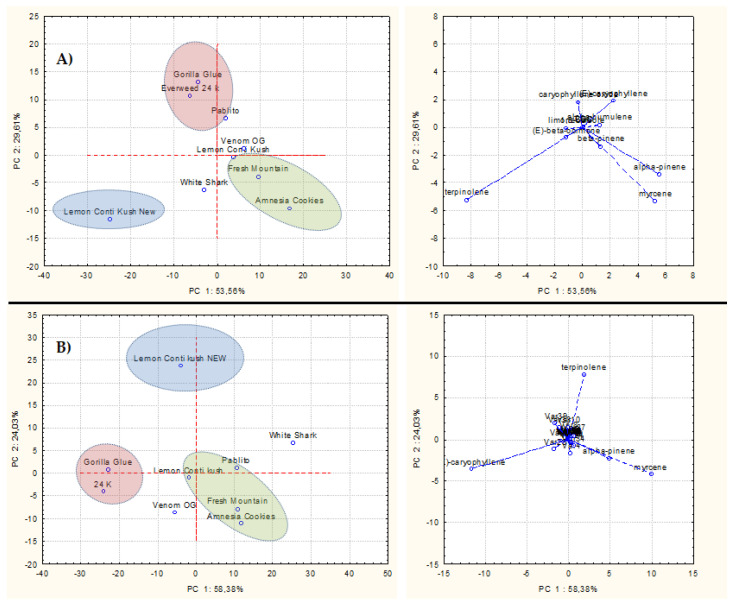
(**A**) Representation of PCA score and loading plots reporting hemp varieties and components found in the 9 EOs. (**B**) Representation of PCA score and loading plots depicting hemp varieties and volatiles extracted by SPME.

The first PCA (Figure 4A) was performed to identify the correlation groups for terpene distribution in EOs, based on the GC-FID analysis results. The plots reported 53.56% of data variability on the first principal component (PC 1), and 29.61% on the second one (PC 2). The variance was caused mainly by terpinolene and, to a minor extent, by α-pinene and myrcene on PC 1, and by (*E*)-caryophyllene and caryophyllene oxide on PC 2. Specifically, the Lemon Conti Kush New cultivar was distinguishable for its high content of terpinolene, while Fresh Mountain and Amnesia Cookies were differentiated by the significant presence of α-pinene and myrcene. The other varieties belonged to the third correlation group, characterized by the sesquiterpenes prevalence.

Another PCA (Figure 4B) was conducted on volatiles obtained by SPME of UM and confirmed the clustering of the first PCA conducted on EOs. In fact, data variability, accounting for 58.38% along the first PC and 24.03% along the second one, was again produced by terpinolene, α-pinene, and myrcene among the monoterpenes, and by the sesquiterpene (*E*)-caryophyllene. Thus, the nine varieties so grouped reflected the trend observed in the first PCA.

**Figure 5:**
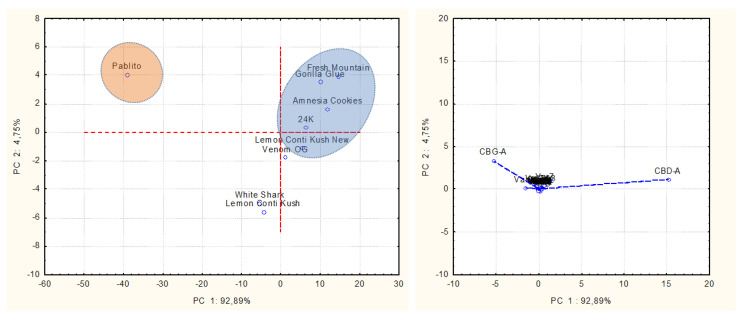
Representation of PCA score and loading plots reporting hemp varieties and their contents of terpenes, polyphenols, and cannabinoids.

The third PCA was carried out on terpenes, polyphenols, and cannabinoids fractions simultaneously (Figure 5), using data related to the dry biomass weight. In this case, PC 1 and PC 2 showed 92.89% and 4.75% variability, respectively. The data distribution was affected especially by CBDA on the first PC and CBGA on the second PC. The Pablito variety was recognized as the one with the highest content of CBGA, and, on the other hand, the CBDA-rich cluster included most of the studied cultivars, namely, Fresh Mountain, Gorilla Glue, Amnesia Cookies, 24 K, and Lemon Conti Kush New.

**Figure 6:**
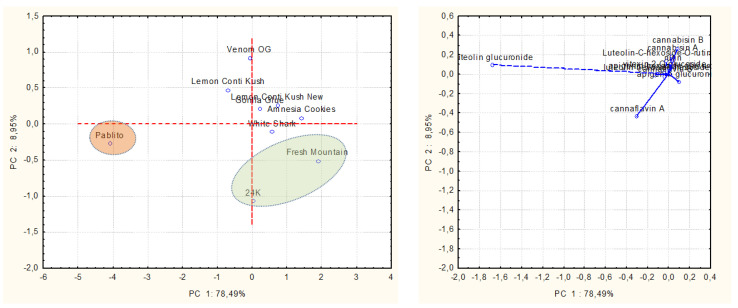
Representation of PCA score and loading plots reporting hemp varieties and the main polyphenols in UM.

The last PCA focused exclusively on the polyphenolic profiles of the nine commercial varieties UM (Figure 6). The plots represented 78.49% of the variation in PC 1 and 8.95% in PC 2. On the first PC, the data variability was influenced mostly by luteolin glucuronide, which was predominant in the Pablito variety; on the second PC, cannflavin A emerged as the main phenolic compound in 24 K and Fresh Mountain.

In summary, the nine hemp commercial varieties were clustered, through PCA analyses, based on volatiles, phenols and cannabinoids content. Regarding the aromatic profile, apart from Lemon Conti Kush New, characterized by terpinolene predominance, the cultivars could be classified as α-pinene/myrcene-rich and (*E*)-caryophyllene-rich chemotypes. Concerning polyphenols and cannabinoids, Pablito was one of the most interesting varieties, because it was marked by the highest concentration of CBGA and luteolin glucuronide. For this reason, it is differentiated from the other cultivars, in which CBDA was prevalent.

### 2.6. Micromorphological Analysis

The pistillate flowers of *C. sativa* are grouped into pairs in crowded, short pauciflore inflorescences at the axillae or terminals of branches. The flower, subtended by a bract, consists of one unilocular ovary and of two elongated, hairy stigmas. A hood-shaped bracteole surrounds the base of the ovary, a typical characteristic of the family Cannabaceae.

The micromorphological survey on the investigated cultivars involved bracts, bracteoles, and inflorescence axes. Within the same variety, the *indumentum* features proved consistent across the replicates, as regards trichome morphotypes, distribution pattern, and density on the examined plant parts.

**Figure 7:**
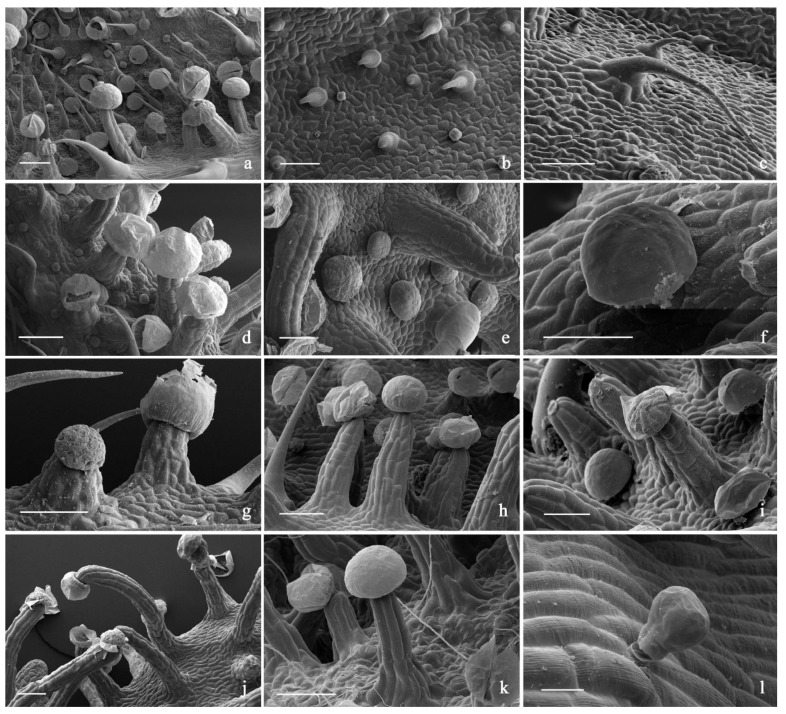
SEM micrographs showing the trichome morphotypes in the investigated *C. sativa* varieties. (**a**) General view of the bract abaxial surface with hair-like lithocysts, capitate-stalked, capitate-sessile and bulbous trichomes; (**b**) short hair-like lithocysts; (**c**) long hair-like lithocysts; (**d**) particular with groups of capitate-stalked and bulbous trichomes; (**e**,**f**) capitate-sessile trichomes; (**g**–**k**) capitate-stalked trichomes with diverse elongation degree of the pseudo-stalk; (**l**) bulbous trichome. *Scale bars = 100 µm (**a**–**k**); 20 µm (**l**)*.

**Figure 8:**
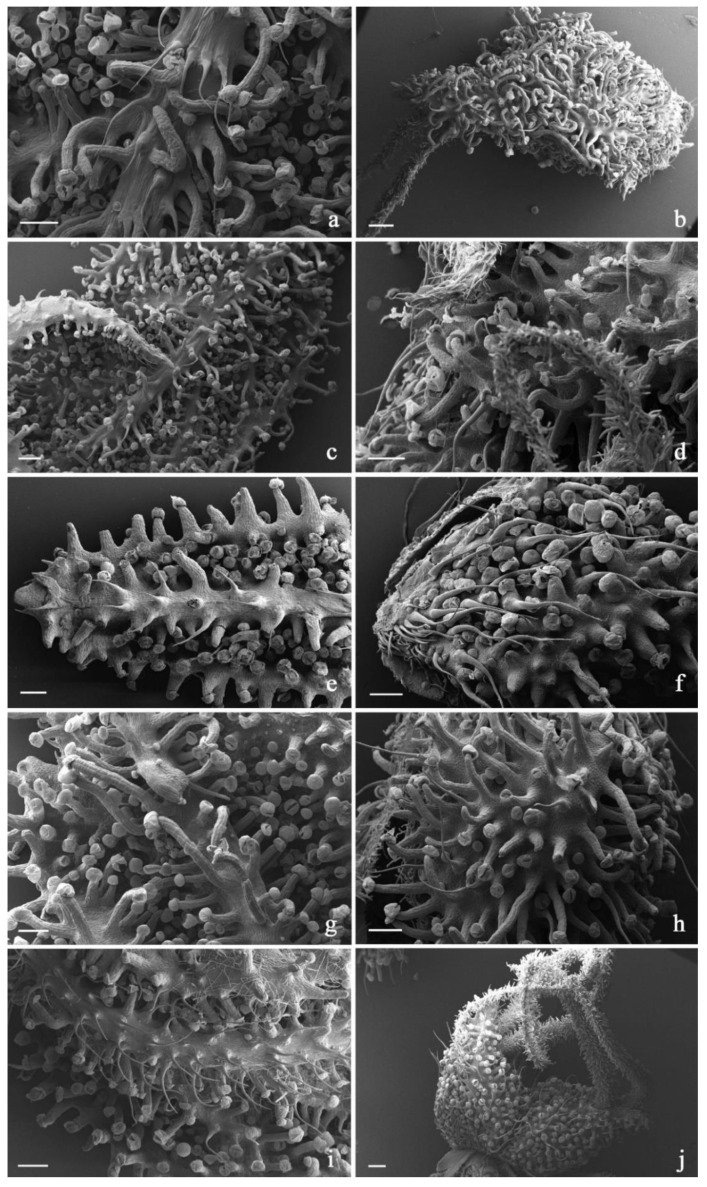
SEM micrographs showing the trichome distribution pattern on bracts and bracteoles of the investigated *C. sativa* varieties. (**a**,**b**) 24 K hemp variety: abaxial surfaces of bract (**a**) and bracteole (**b**); (**c**,**d**) Gorilla Glue hemp variety: abaxial surfaces of bract (**c**) and bracteole (**d**); (**e**,**f**) Lemon Conti Kush hemp variety: abaxial surfaces of bract (**e**) and bracteole (**f**); (**g**,**h**) Fresh Mountain hemp variety: abaxial surfaces of bract (**g**) and bracteole (**h**); (**i**,**j**) Amnesia Cookies hemp variety: abaxial surfaces of bract (**i**) and bracteole (**j**). *Scale bars = 200* *µm (**a**,**c**–**i**); 250* *µm (**b**,**j**)*.

**Figure 9:**
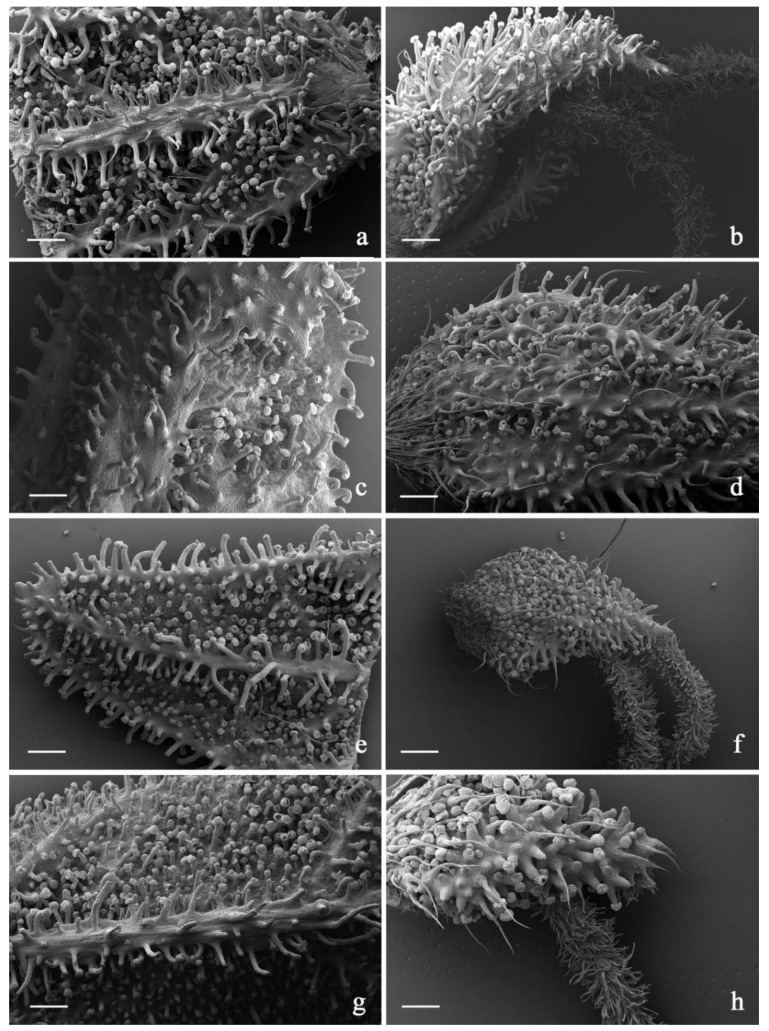
SEM micrographs showing the trichome distribution pattern on bracts and bracteoles of the investigated *C. sativa* varieties. (**a**,**b**) Pablito hemp variety: abaxial surfaces of bract (**a**) and bracteole (**b**); (**c**,**d**) White Shark hemp variety: abaxial surfaces of bract (**c**) and bracteole (**d**); (**e**,**f**) Venom OG hemp variety: abaxial surfaces of bract (**e**) and bracteole (**f**); (**g**,**h**) Lemon Conti Kush New hemp variety: abaxial surfaces of bract (**g**) and bracteole (**h**). *Scale bars = 500 µm (**a**–**g**); 200 µm (**h**)*.

In all the investigated hemp varieties, the plant epidermis was densely covered by an *indumentum* composed of diverse trichome morphotypes (Table 10; Figure 7, Figure 8 and Figure 9).

Hooked hair-like lithocysts were observed on the bract, bracteole, and inflorescence axis surfaces. They were simple and unicellular with an acute apex; the cell diameter was progressively smaller moving from the base to the apex (Figure 7). The cuticle was smooth in Venom OG (Figure 7), and characterized by micropapillae, sometimes lacking on the basal cells, in all the other examined varieties. The overall length of this hair kind appeared variable, being lower on the interveinal regions of bracts and bracteoles and higher along the vein systems. Their distribution patterning and the compositions of the cystoliths have been used in the past in the forensic identification of marijuana [58]. 

In previous literature contributions, glandular trichomes of various morphotypes have been defined under diverse, controversial terms over time [6,9,59,60,61]. Therefore, an update of trichome terminology would be highly desirable to redefine the gland morphotypes. We decided, however, to adopt the current terminology and, consistently with the critical considerations that appeared in the paper by Casiraghi et al. [60], recognized two main trichome groups. 

The first was capitate, with a head made up of 8–16 cells arranged in a single disc and a multiseriate stalk composed of four to eight cell rows. The secretory head was surrounded by a broad storing chamber, giving to the apex of each trichome a spherical shape. Cuticular rupture is often observed in SEM micrographs, in the form of a detached cap or following a horizontal line of apparent fragility in the diametrical region of the head. The stalk was variable in length due to the diverse elongation degree of the epidermal multiseriate stalk (pseudo-stalks) supporting it. Therefore, the so-called capitate-stalked glands and capitate-sessile glands were grouped together. They invariably co-occurred on the inflorescence axis, on the bracts and especially on bracteoles in all the investigated varieties. The second group was bulbous, with a uni- or bicellular head, a short, biseriate stalk and a two-foot cell lying at the level of the epidermis [59]. We recorded their distributions on the surfaces of all the examined plant parts. As a whole, neither the density rate nor the distribution pattern of the different types of trichomes represented features with diagnostic value for varietal recognition (Table 10). However, the diverse elongation degrees and the variable diameters of the pseudo-stalks of the capitate-stalked trichomes appeared as microcharacters useful in the recognition of some examined cultivars. Indeed, in Lemon Conti Kush and Lemon Conti Kush New, the pseudo-stalk appeared typically shorter and wider at the base in comparison to the other examined varieties. 

**Figure 10:**
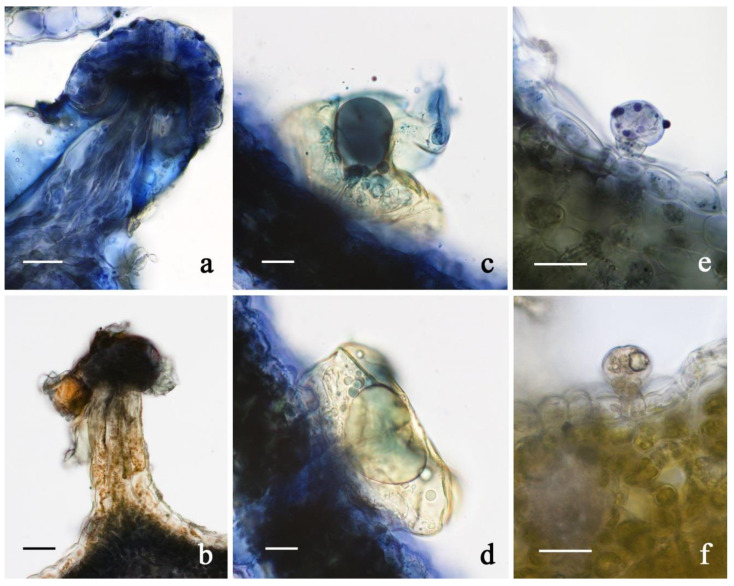
LM micrographs showing the results of the histochemical investigation on the glandular trichomes in the investigated *C. sativa* varieties. (**a**,**b**) Capitate-stalked trichome: Nadi reagent (**a**), Ferric Tricloride (**b**); (**c**,**d**) capitate-sessile trichome: Nadi reagent; (**e**,**f**) bulbous trichome: Nadi reagent (**e**), Ferric Tricloride (**f**). *Scale bars = 20 µm*.

Due to the dense *indumentum* and the overall small sizes of the bulbous hairs, histochemical observation under light microscope mostly involved capitate glands. Copious secretory products fully covering the heads and the stalks of capitates were observed. They were mainly composed of terpenes, both for capitate-stalked and capitate-sessile hairs, as indicated by the intense positive responses to the NADI reagent, with a minor polyphenolic fraction, as suggested by the green-brownish colorations after the application of the Ferric Tricloride stain (Figure 10). The bulbous hairs occasionally exhibited faintly positive responses to terpenes and polyphenols. Cannabinoid production, however, takes place mainly in the capitate trichomes, especially the stalked ones, as was largely confirmed by gas–liquid chromatographic evidence, by the identification of the candidate biosynthetic genes [6], and by CARS microscopy [10].

## 3. Materials and Methods

### 3.1. Origin of the Commercial Varieties

The 9 investigated commercial varieties of *C. sativa*, namely, 24 K, Gorilla Glue, Lemon Conti Kush, Lemon Conti Kush New, Fresh Mountain, Amnesia Cookies, Pablito, White Shark, and Venom OG, were provided by the farm Everweed of G.Di Vietri & C. SS, sited in the national park of Monti Sibillini, in the municipality of Amandola, district of Ascoli Piceno, central Italy (GPS coordinates: 42°58′42.6″ N, 13°24′19.3″ E). They were obtained by crossbreeding between EU-approved cultivars, which has not been fully disclosed due to potential patent protection. Specifically, 24 K and Gorilla Glue derived from male inflorescences of Carmagnola CS; Lemon Conti Kush, Fresh Mountain and Amnesia Cookies originated from Kompolti male inflorescences; Pablito was generated from Santhica 70. In addition, White Shark and Venom OG were produced by replanting 24 K seeds; Lemon Conti Kush New was obtained from Lemon Conti Kush seeds. Voucher specimens of the 9 hemp varieties were stored in the *Herbarium Camerinensis* of the School of Bioscience and Veterinary Medicine, University of Camerino.

### 3.2. Growing and Harvesting Conditions

Hemp plants were cultivated in semi-hilly fields belonging to the farm Everweed. After 18 h of light at the vegetative stage, the agamic reproduction with cuttings was carried out. The cuttings were then positioned in a hydroponic greenhouse, where they were left to take root. The plants were transferred to the ground, and they grew to about 60 cm in height. Drip irrigation and NPK macronutrients were employed, without using pesticides, herbicides, and chemical fertilizers. The inflorescences were harvested from September to the second week of October 2020. Larger leaves were then removed, and the plants were air-dried in the dark, by means of fans and dehumidifiers. After slow drying, flowers were separated from branches, and smaller leaves were mechanically and, if necessary, manually eliminated. The product (untreated material, UM), represented by female inflorescences of the 9 varieties, was placed in plastic bags under vacuum and stored in the dark, until use.

### 3.3. Hydrodistillation

In order to obtain the EOs, 200 g of UM for each variety were subjected to hydrodistillation in a 10 L round flask, filled with 6 L of distilled water. UM was left to soak for 30 min before extraction. A Falc MA heating mantle (Falc Instruments, Treviglio, Italy), and a Clevenger-type apparatus were employed for the process, which was carried out for 5 h. The provided EOs were separated from the aqueous layer and collected in glass vials, to be stored at 4 °C until further analysis. The EOs yields were calculated on a dry matter basis (*w*/*w*).

Along with EOs, two other hydrodistillation products were recovered, namely, the water and plant biomass remaining in the round flask. The aqueous residues were filtered with filter paper and maintained at −20 °C, while the deterpenated material was dried for 24 h at 60 °C within a Biosec desiccator (Tauro Essiccatori, Vanzo Nuovo, Vicenza, Italy), and stored at room temperature in the dark for the following analyses.

### 3.4. Analysis of the Volatile Fraction

#### 3.4.1. GC-FID Analysis of EOs

The quantified EO marker compounds were α-pinene, β-pinene, myrcene, limonene, 1,8-cineole, (*E*)-β-ocimene, terpinolene, (*E*)-caryophyllene, α-humulene, caryophyllene oxide, CBD and THC. Their analytical standards, provided by Sigma Aldrich (Milan, Italy), were injected to build the calibration curves in the range 0.005–10 mg/mL. The EOs obtained from the 9 commercial hemp varieties were diluted 1:100 in analytical-grade *n*-hexane; 0.5 μL of this solution was analyzed in split mode (1:30), by employing an HP-5 coated capillary column (HP-5, 30 m l., 0.32 mm i.d., 0.25 μm f.t., Agilent Technologies), placed in an Agilent 6850 Gas-Chromatograph (GC). A generator PGH2-250 (DBS Analytical Instruments, Vigonza, Italy) was used to produce hydrogen flowing at 3.7 mL/min. The injector temperature was set at 300 °C, while that of the GC oven was programmed as follows: 60 °C for 3 min, then 350 °C at 25 °C/min for 1 min. The FID detector temperature was 360 °C, and the hydrogen and air flow were 40 and 400 mL/min, respectively.

#### 3.4.2. GC-MS Analysis of EOs

The qualitative chemical compositions of the EOs from the 9 varieties were evaluated through an Agilent 8890 GC, with a single quadrupole Agilent 5977B Mass Spectrometer (MSD) (Santa Clara, CA, USA), and a PAL RTC 120 autosampler (CTC Analytics AG, Zwingen, Switzerland). The non-polar HP-5MS (5% phenylmethylpolysiloxane; length: 30 m, 0.25 mm i.d., 0.25 µm f.t.) and the polar DB-WAX (polyethylene glycol; length: 60 m, 0.25 mm i.d., 0.25 μm f.t.) columns were employed as stationary phases. Helium (He) flow rate was 1 mL/min. The oven temperature programs were set as below: for the HP-5MS column, 60 °C for 5 min, then up to 220 °C at 4 °C/min, and on to 280 °C at 11 °C/min, for 15 min, and finally to 300 °C at 15 °C/min for 0.5 min; for the DB-WAX column, 60 °C for 5 min, increased to 220 °C at 4 °C/min, and later to 250 °C at 11 °C/min, for 15 min. The EOs were diluted 1:100 in *n*-hexane (LC-MS) and the injection was done in split mode (1:200). Data were acquired in SCAN mode (40–400 *m/z*) and analyzed through MSD ChemStation software (Agilent, Version G1701DA D.01.00), using the NIST Mass Spectral Search Program for the NIST/EPA/NIH EI and NIST Tandem Mass Spectral Library v. 2.3. A mix of *n*-alkanes (C_8_-C_30_, Supelco, Bellefonte, CA, USA) was injected to calculate linear retention indices (RI), and EO constituents were identified by checking the correspondence between their RI and mass spectra (MS) and those of commercial libraries, in particular ADAMS in the case of the analysis performed with the HP5-MS column, and NIST 17 when the DB-WAX column was used [62,63]. Relative abundance (peak area percentages) was obtained by normalization without using correction factors.

#### 3.4.3. Chiral GC-MS Analysis of EOs

The separation of the enantiomeric pairs of α-pinene, β-pinene, limonene, linalool, (*E*)-caryophyllene and caryophyllene oxide in the 9 EOs was achieved with an Agilent HP 20β capillary column (20% β-cyclodextrin, length: 30 m, 0.25 mm i.d., 0.25 μm f.t.). The employed GC-MS system, along with the EOs dilution and injection mode, were the same as those used in the previous analyses (Section 3.4.2). The oven temperature was set at 50 °C, which was then raised to 220 °C at 2 °C/min for 1 min. The injector temperature was 250 °C, while the ionization source and quadrupole temperatures were set at 230 °C and 150 °C, respectively. The analytical standards (Sigma-Aldrich) of (+)-α-pinene, (−)-α-pinene, (−)-β-pinene, (+)-limonene, (−)-linalool, (−)-(*E*)-caryophyllene and (−)-caryophyllene oxide were injected as reference compounds.

#### 3.4.4. SPME-GC-MS Analysis of UM

To analyze the volatile composition of UM, an SPME device from Supelco (Bellefonte, PA, USA) with 1 cm fiber coated with 50/30 μm DVB/CAR/PDMS (divinylbenzene/carboxen/polydimethylsiloxane) was used. The operative conditions for the sampling were the following: equilibration time of 30 min, and sampling time of 60 min at 35 °C. Lastly, the SPME fiber was inserted into the injector of the GC-MS system, maintained at 250 °C and operating as below. A gas chromatograph equipped with a FID and coupled with a mass spectrometer (Clarus 500 model Perkin Elmer-Waltham, MA, USA) was used. The capillary column was a Varian Factor Four VF-1, and the optimized temperature program was the following: from 70 °C to 120 °C at 6 °C/min; from 120 °C to 220 °C at 7 °C/min and held for 10 min. The components were identified by comparison between their calculated linear retention indices (LRIs) and those relating to a mix of *n*-alkanes. Furthermore, the matching of their mass spectra against commercial libraries (NIST) was performed. All analyses were conducted in triplicate and the results were expressed as average percentages calculated by peak area normalization from GC-FID chromatograms, without the use of an internal standard or correction factors.

### 3.5. Spectrophotometric Analysis of LE and DM

#### 3.5.1. Samples Treatment

The frozen remaining water after EO distillation was freeze-dried at − 54 °C and 0.05 mbar, through a BUCHI Lyovapor™ L-200 freeze-dryer (Büchi Labortechnik AG, Flawil, Switzerland). The lyophilized extracts (LE) were ground in a mortar and the obtained powders were maintained at 4 °C until further analyses.

#### 3.5.2. Total Polyphenols Content

The total polyphenols content (TPC) in LE was measured by applying the Folin–Ciocalteu method [64], with little variation. Briefly, 0.5 mL of aqueous solutions of LE (1 mg/mL), after being added to 2.5 mL of Folin–Ciocalteu reagent solution (diluted 10 times in water) and 7 mL of 7.5% Na_2_CO_3_ solution, were stored for 2 h at room temperature in the dark. The spectrophotometric assay was carried out with a Cary 8454 UV-Vis (Agilent Technologies, Woburn, MA, USA) at 735 nm. The calibration curve of gallic acid was employed in order to determine the TPC, which was reported as the mean of two measurements, and indicated as mg of gallic acid equivalents (GAE) per g of dry extract (DE). The TPC was evaluated also for the dried deterpenated material (DM). In this case, 1 g of DM was extracted in an ultrasound bath with 10 mL of a 50% ethanol aqueous solution, and 0.5 mL of the supernatant was subjected to the spectrophotometric test, following the same previous procedure. The results were expressed as mg GAE per g of dry hemp (DW).

#### 3.5.3. Total Flavonoids Content

The total flavonoids content (TFC) determination was performed by following the procedure by Chen et al. [65], with few modifications. More precisely, 0.5 mL LE solutions in water at a concentration of 1.5 mg/mL were treated with 0.15 mL of NaNO_2_ (0.5 M), with the following addition with stirring of 3.2 mL of 30% MeOH and 0.15 mL of AlCl_3_·6H_2_O (0.3 M). After adding 1 mL of NaOH (1 M) 5 min later, the absorbance of the mixed solution was evaluated spectrophotometrically at 506 nm. Rutin calibration curve (100–1000 ppm) was used, and the results are given as mg of rutin equivalents (RE) per g of DE. DM was extracted with a 50% ethanol solution in water for TFC determination, operating in the same way as the TPC analysis of DM. The spectrophotometric assay was carried out as already described in this section and the obtained data were reported as mg RE per g of DW.

#### 3.5.4. Radical Scavenging Activity

The antioxidant activity was determined in compliance with Mustafa et al. [64], by using 2,2-diphenyl-1-picrylhydrazyl (DPPH) free radical. Specifically, 4.5 mL of DPPH in EtOH (0.1 mM) were added to the aqueous solutions of LE (0.5 mL), to be then kept at room temperature for 30 min in the dark. The analysis was performed at 517 nm, with the same spectrophotometer employed for TPC and TFC evaluation. The radical scavenging activity was expressed, referring to the trolox calibration curve, as mg of trolox equivalents (TE) per g of DE. For DM, the same ethanol/water extracts were prepared, as for the two previous spectrophotometric tests, and then they were analyzed as reported in the present section. The antioxidant capacity was measured as mg TE per g of DW. 

### 3.6. ^1^H-NMR Analysis of UM and DM

As a preliminary characterization approach, UM and DM were subjected to ^1^H-NMR analysis. In detail, 100 mg of ground samples were extracted with 800 µL of CDCl_3_, sonicated for 10 min and centrifuged. The liquid was then collected in an NMR tube, while the plant material was dried under a nitrogen flow and then re-extracted with deuterated methanol and water, sonicated for 10 min and centrifuged. In these cases, the liquid fractions were collected in NMR tubes and analyzed. The spectra were acquired using a Bruker Ultrashield Plus 400 MHz spectrometer.

### 3.7. HPLC-DAD-MS^n^ Analysis of LE, DM and UM

An Agilent 1260 chromatograph with an autosampler and a diode array detector (DAD), interfaced with a Varian MS 500 ion trap mass spectrometer, was employed to quantify cannabinoids and phenolic compounds in the LE, DM, and UM of the 9 hemp commercial varieties. For the analysis of flavonoids and minor compounds, the column was an Agilent Eclipse XDB C18 (3.0 m × 150 mm × 3.5 μm), and the mobile phase was represented by a mixture of 1% formic acid in water (A) and acetonitrile (B). At the beginning, the gradient was 95% A, and in 30 min reached 100% B, with a flow rate of 0.4 mL/min. Data were collected by DAD in the λ range of 200–400 nm. The mass spectrometer was provided with an electrospray ion (ESI) source, which was employed in negative ion mode. The MS parameters were the following: spray chamber temperature, 45 °C; needle voltage, 4700 V; capillary voltage, 85 V; RF loading, 80%; nebulizing gas pressure, 25 psi (nitrogen); drying gas pressure, 15 psi; drying gas temperature, 300 °C. Spectra were acquired in the 50–1000 *m/z* range. Chromatograms were acquired from the turbo data depending on the scanning (TDDS) mode, allowing the generation of fragmentation spectra for the most intense ionic species. For the analysis of cannabinoids, an Agilent XDB (4.6 m × 250 mm × 5.0 μm) was used as the stationary phase. The gradient of elution was performed using water 1% formic acid (A) acetonitrile (B) and methanol (C). The gradient started with 30% A and 70% B, and in 20 min arrived at 70% B and 30% C; in 23 min it reached 100% C and stayed isocratic up to 33 min. Data were detected with DAD in the λ range of 200–400 nm. ESI-MS spectra were collected in positive ion mode for the neutral cannabinoids, and in negative ion mode for the acidic forms. Compounds were identified based on *m/z* values and retention times, and by comparison with authentic standards. For quantitative purposes, standard solutions in concentration from 0.1 to 100 μg/mL were set up to develop the calibration curves. For this purpose, CBD, cannabidiolic acid (CBDA), THC, and δ-9-tetrahydrocannabinolic acid (THCA) were used for cannabinoids’ quantification, while rutin and quercetin-3-O-glucoside were selected for phenols content determination. For the analyses, LE, DM and UM samples were finely ground and about 200 mg were weighed and extracted with 25 mL of MeOH/H_2_O 70:30. The extracts were sonicated in an ultrasound bath for 15 min and centrifuged, and then the supernatants were taken and inserted into HPLC vials.

### 3.8. Multivariate Statistical Analysis of EOs and UM

Principal Component Analysis (PCA) was executed on the compositions of EOs and UM of the 9 commercial varieties, using STATISTICA software v. 7.1 (Stat Soft Italia S.r.l., Vigonza, Italy). GC-FID (Section 2.1.2) and SPME (Section 2.1.5) results for terpenes, and HPLC data (Section 2.4) for polyphenols and cannabinoids, were employed to build the score and loading plots (missing values were replaced with 0.001).

### 3.9. Micromorphological Analysis

For each hemp cultivar, a micromorphological survey on female inflorescences (bracts, bracteoles and inflorescence axes) was carried out by means of light microscopy (LM) and scanning electron microscopy (SEM) in order to document the features of the glandular indumentum. The samples were collected at comparable anthesis phenological stages. A minimum of ten replicates per each plant part were studied to assess the variability in the micromorphological features. Referring to the trichome distribution, we qualitatively evaluated it using the following symbols: (+) present in all the replicates; (++) abundant in all the replicates with a distribution on the whole organ surface.

#### 3.9.1. Light Microscopy (LM)

The fresh examined plant parts were preliminarily observed by LM using hand-cut sections. The following histochemical dyes were used to characterize the chemical nature of the secretory products of the glandular trichomes: Toluidine Blue as a general dye [66], Nadi reagent for terpenes [67], Alcian Blue for mucopolysaccharides [66], and Ferric Trichloride for polyphenols [68]. Control stainings were simultaneously carried out. Observations were performed under a Leitz DM-RB Fluo optical microscope equipped with a Nikon digital camera.

#### 3.9.2. Scanning Electron Microscopy (SEM)

Small hand-prepared segments of each plant part were FAA-fixed for 7 days, dehydrated in ascending ethanol series up to absolute, critical-point-dried, mounted on stubs and carbon gold-coated. Observations were performed under a Zeiss^®^ EVO MA15 SEM operating at 10 kV at the Interdepartmental Center for Electron Microscopy and Microanalysis Services (M.E.M.A.) of the University of Florence (Florence, Italy).

## 4. Conclusions

The findings of this work highlight new opportunities for the working area on hemp. In addition to the most common applications and uses of hemp, its aerial parts, in particular the inflorescences, can be exploited to obtain essential oil by hydrodistillation, a niche and valuable product to be employed especially in the area of perfumes, and in agriculture as natural pest-control agent, though new applications remain to be identified. Concerning the residual fractions after hydrodistillation, the aqueous residue was found to be rich in phenolic compounds, thus it can be a source of antioxidant constituents used as additive in food and cosmetics. In addition, the deterpenated material has been proven to contain a significant amount of decarboxylated cannabinoids, this being a valuable starting material for the extraction of such compounds for the pharmaceutical market. From a phytochemical point of view, the nine commercial hemp varieties showed significant differences in terms of volatile profiles and, to a minor extent, the major cannabinoids. Interestingly, Lemon Conti Kush New was characterized by high levels of the monoterpene terpinolene, while Pablito was distinguishable from the others for the high levels of cannabigerol. In terms of polyphenols, the latter was the only one showing high contents of luteolin-3-glucuronide, while 24 K and Freh Mountain contained a high level of cannflavin A. The micromorphological and histochemical survey on the examined hemp commercial varieties allowed us, for the first time, to sketch a link between the hair morphotypes and their phytochemical profiles. However, neither the trichomes’ density rate, nor their distribution patterns on inflorescences, represented features with diagnostic value for varietal differentiation. Only the smooth cuticle of the hooked hair-like lithocysts and the shorter pseudo-stalks of the capitate-stalked trichomes proved valuable for the recognition of Venom OG and Lemon Conti Kush/Lemon Conti Kush New varieties, respectively. 

## Figures and Tables

**Table 1:** GC-FID analysis results for the 9 commercial varieties’ EOs and their yield values (%).

Compound	Hemp Variety								
	White Shark	Lemon Conti Kush New	Lemon Conti Kush	Venom OG	Pablito	24 K	Fresh Mountain	Amnesia Cookies	Gorilla Glue
(g/100 g)									
α-pinene	8.53	3.24	11.48	21.19	8.35	0.73	9.32	21.16	0.74
β-pinene	3.59	2.78	4.67	4.79	3.21	0.68	3.66	7.45	0.82
myrcene	20.28	11.14	17.13	10.57	12.56	8.12	27.46	29.23	7.16
limonene	9.17	5.97	3.38	4.28	3.30	4.18	4.10	2.20	6.79
1,8-cineole	0.07	0.13	0.04	0.07	0.03	0.02	0.04	0.04	0.02
(*E)-*β-ocimene	0.10	4.51	0.16	0.05	0.06	0.03	0.02	0.02	0.03
terpinolene	9.51	30.47	1.33	1.65	0.52	3.69	0.25	0.18	0.27
(*E*)-caryophyllene	10.79	10.40	11.91	17.19	18.15	18.12	19.70	18.91	18.94
humulene	2.96	3.04	4.46	4.64	6.82	5.35	5.51	8.57	4.86
caryophyllene oxide	3.52	3.34	3.88	5.60	7.97	8.00	1.28	3.93	6.62
CBD	5.54	2.91	4.26	5.25	3.13	4.02	3.86	2.30	3.06
Δ-9-THC	0.13	0.09	0.10	0.13	0.12	0.12	0.12	0.20	0.20
Yields (%)	0.64	1.51	1.03	0.86	0.49	1.08	1.23	1.81	1.33

**Table 2:** GC-MS analysis results for the 9 commercial varieties EOs.

								Variety							
						Pablito	White Shark	Gorilla Glue	24 K	Fresh Mountain	Venom OG	Lemon Conti Kush	Lemon Conti Kush New	Amnesia Cookies	
N	Component ^a^	RI ^b^ HP-5MS	RI ^c^ lit. apol.	RI ^d^ DB-WAX	RI ^e^ lit. polar	% HP-5MS									ID ^f^
1	heptanal	901	901	1187	1184	0.9	0.3	0.2	0.2	0.2	0.3	0.6	0.1	tr ^g^	RI,MS
2	α-thujene	926	924	1028	1025		0.1		0.2			tr	0.3	tr	RI,MS
3	α-pinene	932	933	1026	1027	9.0	11.3	0.7	0.2	11.0	23.3	12.5	3.5	24.6	Std
4	camphene	947	946	1071	1063	0.1	0.3	0.1	0.2	0.2	0.4	0.2	0.1	0.3	Std
5	β-pinene	975	974	1114	1112	3.0	4.2	0.7	0.2	4.0	5.0	4.8	2.7	8.2	Std
6	myrcene	990	988	1164	1165	10.7	21.5	6.6	0.2	26.0	9.7	14.9	9.5	27.1	Std
7	α-phellandrene	1004	1002	1169	1167	tr	0.5			tr	tr	tr	1.3	tr	Std
8	δ-3-carene	1009	1008	1153	1152		0.4					tr	1.0		Std
9	α-terpinene	1016	1014	1184	1193	tr	0.4		0.1	tr	tr	tr	1.3	tr	Std
10	limonene	1027	1024	1203	1193	2.7	10.2	6.8	4.6	3.9	3.9	2.8	5.4	1.8	Std
11	1,8-cineole	1030	1026	1217	1212	0.1	tr		1.5		tr		0.1	0.2	Std
12	(*Z*)-β-ocimene	1038	1032				tr				tr		tr		Std
13	(*E*)-β-ocimene	1048	1044	1253	1252	tr	tr					tr	4.0		Std
14	γ-terpinene	1058	1054	1250	1257	tr	0.3	tr	0.3		tr	0.1	1.0	tr	Std
15	terpinolene	1087	1086	1288	1282	0.3	11.8	0.3	0.4	0.2	0.1	1.0	30.2	0.1	RI,MS
16	linalool	1099	1095	1547	1549	0.2	1.0	0.9	2.9	0.1	1.0	0.4	0.4	tr	Std
17	*endo*-fenchol	1112	1114	1589	1582	0.2	0.8	1.7	1.0	0.8	0.7	0.6	0.4	tr	RI,MS
18	*trans*-pinene hydrate	1120	1119			0.1	0.5	1.1	0.6	0.5	0.4	0.4	0.3	tr	RI,MS
19	borneol	1164	1165	1711	1701	0.1	0.2	0.4	0.3	0.2	0.3	0.2	0.1	tr	Std
20	α-terpineol	1189	1186	1703	1698	0.3	1.0	1.4	1.3	0.6	0.7	0.4	1.0	0.1	Std
21	hexyl isobutanoate	1192	1191					0.1			tr	0.3			RI,MS
22	sativene	1393	1390					0.1		tr		0.1	tr		RI,MS
23	α-*cis*-bergamotene	1416	1411	1577	1577		0.1	0.1	0.2	tr	0.1	0.1			RI,MS
24	(*E*)-caryophyllene	1420	1417	1612	1612	15.5	11.4	17.6	19.9	16.0	17.1	10.7	8.2	15.3	Std
25	α-*trans*-bergamotene	1436	1432	1594	1583	0.2	1.0	1.3	1.4	0.4	0.7	0.9	0.1	0.1	RI,MS
26	α-guaiene	1439	1439					tr		2.8					RI,MS
27	α-humulene	1455	1452	1685	1667	6.5	3.0	4.7	6.3	4.7	4.9	4.1	2.4	7.6	Std
28	(*E*)-β-farnesene	1457	1454	1668	1674	0.1	1.1	1.2	1.5	0.2	0.6	0.8	0.1	tr	Std
29	*allo*-aromadendrene	1462	1458			0.1		tr	0.2	tr	tr	0.2	tr	tr	RI,MS
30	γ-muurolene	1478	1478					0.1		0.1		0.1			RI,MS
31	γ-curcumene	1480	1481					0.1	tr						RI,MS
32	β-selinene	1487	1489	1736	1734	0.8	0.2	0.2		0.6	0.1	0.5	0.6	1.3	RI,MS
33	valencene	1494	1496			0.2		0.4			0.1			0.1	RI,MS
34	α-selinene	1496	1498	1740	1737	0.7	0.2	0.5	0.1	0.8	0.1	0.8	0.7	1.1	RI,MS
35	β-dihydro-agarofuran	1501	1503			0.1	tr	0.1	0.1		0.1	tr	tr	tr	RI,MS
36	α-bulnesene	1507	1509			0.1				6.3					RI,MS
37	β-bisabolene	1509	1505	1734	1727	0.7	0.1	1.4	1.9	1.1	0.1	1.6	0.6	0.1	RI,MS
38	β-curcumene	1512	1514				0.1	0.2	0.2		tr	0.1			RI,MS
39	sesquicineole	1515	1515			tr	0.1	0.1	0.5	0.1	0.2	0.3	tr		RI,MS
40	zonarene	1522	1528			0.1	tr	0.9		0.3	tr				RI,MS
41	selina-4(15),7(11)-diene	1536	1544			0.3	0.1	7.1		3.5	0.1	5.0	3.4	0.1	RI,MS
42	selina-3,7(11)-diene	1543	1538	1796	1791		0.3	12.5		7.8	0.3	8.7	6.4	0.7	RI,MS
43	(*E*)-α-bisabolene	1543	1544	1780	1784	1.7			1.8						RI,MS
44	caryophyllene oxide	1584	1582	2008	2005	0.8	0.2	0.2	0.5	0.6	1.0	0.4	0.1	0.1	Std
45	guaiol	1598	1600	2100	2103	7.5	3.0	5.8	9.7	tr	5.9	3.3	2.1	2.1	RI,MS
46	eudesmol-5-*epi*-7-*epi*-α	1606	1607			0.4	0.1	0.2	0.4		0.2	0.1	0.1	tr	RI,MS
47	10-*epi*-γ-eudesmol	1621	1622	2124	2137	7.6	4.1	7.0	10.2	tr	6.5	4.6	3.1	3.0	RI,MS
48	γ-eudesmol	1633	1630			1.7	0.5	0.8	1.5		1.0	0.5	0.3	0.3	RI,MS
49	β-eudesmol	1651	1649			3.1	1.3	1.8	3.4	tr	2.3	1.1	0.7	0.7	RI,MS
50	α-eudesmol	1654	1652			4.3	2.0	2.8	4.7	0.2	3.2	1.7	1.1	1.1	RI,MS
51	eudesmol-7-*epi*-α	1659	1658			0.8	0.3	0.5	0.9		0.6	0.3	0.1	0.1	RI,MS
52	bulnesol	1668	1670	2201	2196	6.0	2.6	4.4	7.2		3.9	2.7	1.7	1.7	RI,MS
53	α-bisabolol	1684	1685	2215	2219	8.6	0.2	2.7	8.4	4.3	0.5	5.2	1.9	tr	Std
54	eudesm-7(11)-4-ol	1697	1700			tr	tr	0.1		tr	tr	0.3	0.1		RI,MS
55	cannabidiol	2427	2430			0.2	0.6	0.2	0.4	0.5	1.0	0.5	0.1	0.1	Std
56	cannabichromene	2434	2440			tr	tr	tr	tr	tr	tr	tr	tr	tr	RI,MS
	Total identified (%)					96.07	97.51	96.13	95.42	98.13	96.63	93.80	96.47	98.42	

^a^ Order of compounds is according to their elution from the HP-5MS column. ^b^ Linear retention index calculated using a mixture of *n*-alkanes (C_8_–C_30_) with respect to HP-5MS column. ^c^ Retention index values for apolar columns taken from ADAMS library. ^d^ Linear retention index calculated using a mixture of *n*-alkanes (C_8_–C_30_) with respect to the DB-WAX column. ^e^ Retention index values for polar columns taken from NIST 17 library. ^f^ Peak assignment method: Std, comparison with an available analytical standard; MS, MS matching with those stored in ADAMS, WILEY 275, NIST 17 and FFNSC2 libraries; RI, comparison of the calculated RI with those reported in ADAMS, NIST 17 and FFNSC2 libraries. ^g^ tr, traces (% < 0.1).

**Table 3:** Chiral GC-MS analysis results for the 9 commercial varieties EOs.

Variety	Enant. %					
	α-Pinene (−):(+)	β-Pinene (+):(−)	Limonene (−):(+)	Linalool (−):(+)	(*E*)-Caryophyllene (+):(−)	Caryophyllene Oxide (+):(−)
**White Shark**	11.6:88.4	70.9:29.1	93.5:6.5	8.4:91.6	0:100	0:100
**Lemon Conti Kush**	4.9:95.1	78.4:21.6	92.4:7.6	6.9:93.1	0:100	0:100
**Lemon Conti Kush New**	3.2:96.8	66.4:33.6	91.4:8.6	34.8:65.2	0:100	0:100
**Pablito**	1.6:98.5	84.8:15.2	90.4:9.6	12.3:87.7	0:100	0:100
**Fresh Mountain**	7.8:92.2	74.6:25.4	93.0:7.0	0:100	0:100	0:100
**Amnesia Cookies**	2.9:97.1	88.6:11.4	74.1:25.9	28.2:71.8	0:100	0:100
**24 K**	7.0:93.0	0:100	97.4:2.6	2.8:97.2	0:100	0:100
**Venom OG**	4.6:95.4	81.0:19.0	87.9:12.1	0:100	0:100	0:100
**Gorilla Glue**	7.9:92.1	0:100	97.4:2.6	0:100	0:100	0:100

**Table 4:** SPME-GC-MS analysis results for the 9 commercial varieties’ UM.

No	Compound					Variety				
		Lemon Conti Kush	Fresh Mountain	24 K	Gorilla Glue	White Shark	Venom OG	Lemon Conti Kush New	Amnesia Cookies	Pablito
						%				
1	α-thujene					tr		0.2		
2	α-pinene	14.4	10.7	1.5	1.4	12.0	3.6	2.0	18.3	12.4
3	camphene	0.2	0.2	tr	0.1	0.2	0.4		0.2	0.2
4	β-pinene	6.1					6.8		6.4	
5	myrcene	21.3	39.2	18.3	15.0	44.6	31.8	15.9	37.2	35.2
6	α-phellandrene					0.6		0.9	tr	0.3
7	α-terpinene									0.2
8	limonene	3.8	3.8	6.2	8.3	11.0	8.2	5.4	1.8	5.9
9	*p*-cymene					0.1				
10	(*Z*)-β-ocimene				0.1	0.1	1.1	5.5		1.2
11	γ-terpinene					0.3		0.4		0.2
12	*p*-cymenene					0.2				
13	terpinolene	1.7	1.0	5.2	2.7	17.1	1.3	24.1	tr	9.2
14	*cis*-ocimenol									0.2
15	copaene									0.1
16	aristolene								0.1	
17	fenchol	0.1		0.2	0.4	0.1				
18	butanoic acid, hexyl ester	0.2								
19	α-terpineol	0.3	0.1	0.5	0.6	0.4	0.3	0.4		
20	ylangene	0.3						0.1		
21	α-santalene	0.2		0.3	0.4	tr	0.1			
22	*trans*-α-bergamotene			0.9	0.4					
23	β-caryophyllene	25.7	23.1	50.3	43.8	10.9	36.5	22.8	23.0	21.8
24	α-guaiene		3.6				0.4			
25	α-himachalene	2.3	1.7							
26	(*E*)-β-farnesene	0.2		0.4						
27	β-bisabolene			3.2	2.2					
28	α-humulene	7.1	5.1	10.6	7.4	1.9	8.0	5.6	9.0	6.5
29	aromadendrene	0.3		0.2						
30	β-eudesmene	0.6	0.7			0.2			1.3	0.7
31	α-farnesene	0.1	0.1		0.5			0.6		
32	δ-selinene							0.4	0.4	
33	valencene				0.4					
34	δ-guaiene		5.8				0.7		1.1	
35	β-sesquiphellandrene	0.1								
36	δ-cadinene	0.1								
37	*trans*-α-bisabolene	1.9	1.6	2.1			0.3	2.4	0.6	1.1
38	selina-4(15),7-(11)-diene	5.1	0.3		6.3			5.2		1.8
39	selina-3,7(11)-diene	7.6	3.0		10.0	0.1	0.3	7.9	0.3	2.6
40	guaia-3,9-diene									0.4
	Total identified (%)	99.7	100.0	99.9	100.0	99.8	99.8	99.8	99.7	100.0

**Table 5:** TPC, TFC, and antioxidant activity results for the nine commercial varieties’ LE.

Variety	TPC (mg GAE/g DE)	TFC (mg RE/g DE)	DPPH (mg TE/g DE)
**Lemon Conti Kush New**	72.18	46.30	142.33
**Lemon Conti Kush**	54.74	30.00	111.89
**Pablito**	66.28	41.63	135.33
**Fresh Mountain**	40.38	18.15	90.33
**24 K**	65.00	40.37	138.11
**Venom OG**	69.62	42.96	143.00
**Amnesia Cookies**	57.82	25.56	109.67
**White Shark**	62.69	30.78	115.89
**Gorilla Glue**	57.56	31.48	120.78

**Table 6:** TPC, TFC, and antioxidant activity results for the 9 commercial varieties DM.

Variety	TPC (mg GAE/g DW)	TFC (mg RE/g DW)	DPPH (mg TE/g DW)
**Lemon Conti Kush**	15.51	12.58	25.07
**Pablito**	16.31	13.58	36.84
**Fresh Mountain**	16.13	10.33	24.11
**24 K**	23.00	22.00	39.07
**Venom OG**	23.90	20.08	41.67
**Amnesia Cookies**	15.82	8.33	26.62
**White Shark**	18.10	13.58	34.40
**Gorilla Glue**	20.28	16.92	38.62
**Carmagnola CS**	9.54	3.25	13.04
**Santhica**	12.36	4.33	10.22
**Kompolti**	15.10	6.50	15.56

**Table 7:** HPLC-DAD-MS^n^ characterization of flavonoids in UM and DM.

Compound	Variety																	
	White Shark		24 K		Lemon Conti Kush		Lemon Conti Kush New		Pablito		Venom OG		Gorilla Glue		Fresh Mountain		Amnesia Cookies	
	UM	DM	UM	DM	UM	DM	UM	DM	UM	DM	UM	DM	UM	DM	UM	DM	UM	DM
mg/g																		
cannabisin A	0.18	0.02	0.07	0.02	0.25	0.03	0.58		0.06	0.01	0.57	0.02	0.86	0.12	0.03	0.01	0.05	0.08
cannabisin B	0.38	0.12	0.05	0.05	0.12	0.14	1.35		0.23	0.02	0.82	0.08	0.81	0.13	0.02	0.004	0.54	0.12
luteolin-C-hexoside-O-rutinoside	0.72	0.05	0.05	0.05	0.16	0.03	0.63	0.03	0.50	0.03	0.66	0.12	0.51	0.02	0.18		0.38	0.06
rutin	0.53	0.04	0.30	0.02	0.33	0.03	0.33	0.06	0.07	0.11	0.51	0.03	0.27	0.04	0.03	0.03	0.21	0.03
luteolin-hexoside-hexoside	0.16	0.04	0.12	0.04	0.13	0.02	0.06	0.03	0.50	0.02	0.15	0.06	0.04	0.02	0.01		0.18	0.06
vitexin 2″-O-glucoside	0.12	0.03	0.12	0.05	0.09	0.02	0.17	0.04	0.30	0.07	0.29	0.03	0.15	0.02	0.17	0.04	0.05	0.03
apigenin-hexoside-glucuronide	0.20	0.06	0.15	0.03	0.17	0.54	0.06	0.02	0.12	0.03	0.18	0.03	0.13	0.46	0.16	0.04	0.13	0.02
luteolin 7-glucuronide	2.77	0.56	3.11	0.69	4.18	0.12	2.58	0.99	7.26	1.49	3.52	0.30	3.10	0.12	1.43	0.29	2.00	0.30
apigenin 7-glucuronide	0.94	0.27	0.46	0.13	0.67	0.07	0.23	0.20	0.21	0.37	0.02	0.15	0.37	0.07	0.48	0.13	0.63	0.14
cannflavin B	0.89	0.88	0.72	0.75	0.67	0.43	0.36	0.9	0.51	0.56	0.58	0.42	0.33	0.25	0.34	0.51	0.35	0.43
cannflavin A	1.13	1.38	2.08	2.66	0.38	1.92	1.46	2.43	2.15	2.87	0.53	0.74	1.35	1.41	0.97	1.3	0.68	0.16

**Table 8:** HPLC-DAD-MS^n^ characterization of flavonoids in LE.

Compound	Variety								
	White Shark	24 K	Lemon Conti Kush	Lemon Conti Kush New	Pablito	Venom OG	Gorilla Glue	Fresh Mountain	Amnesia Cookies
	(mg/g)								
cannabisin A	0.33	0.39	0.22	0.30	0.57	0.11	0.33	0.33	0.12
cannabisin B	0.71	2.57	2.37	0.35	3.03	3.57	0.30	0.22	0.18
luteolin-C-hexoside-O-rutinoside	0.23	1.18	0.51	0.50	1.48	1.39	2.51	0.22	0.32
rutin	7.93	7.93	8.79	0.35	5.63	12.18	0.75	0.30	0.58
luteolin-hexoside-hexoside	0.21	0.20	0.49	2.35	0.21	0.34	0.21	0.38	0.21
vitexin 2″-O-glucoside	0.29	3.19	3.48	0.63	0.55	5.01	0.40	0.57	0.37
apigenin-hexoside-glucuronide	1.12	0.79	1.43	0.32	1.84	1.21	0.79	0.46	0.32
luteolin 7-glucuronide	0.05	0.30	0.20	0.27	0.22	0.05	0.29	0.34	0.22
apigenin 7-glucuronide	0.22	0.07	0.04	0.27	0.23	0.22	0.31	0.23	0.08
cannflavin B	0.05	0.04	0.08	0.23	0.09	0.16	0.12	0.06	0.20
cannflavin A	0.24	0.19	0.15	0.27	0.12	0.20	0.62	0.20	0.24

**Table 9:** HPLC-DAD-MS^n^ characterization of cannabinoids in UM and DM.

Compound	Variety																	
	White Shark		24 K		Lemon Conti Kush		Lemon Conti Kush New		Pablito		Venom OG		Gorilla Glue		Fresh Mountain		Amnesia Cookies	
	UM	DM	UM	DM	UM	DM	UM	DM	UM	DM	UM	DM	UM	DM	UM	DM	UM	DM
mg/g																		
CBDA	46.12	0.68	59.07	2.34	46.92	1.22	62.45	2.95	20.68	0.35	53.70	1.45	63.52	2.61	67.77	2.44	64.14	2.40
CBD	0.35	1.56	0.74	2.89	0.43	2.38	0.72	3.71	0.16	0.61	0.56	3.25	0.94	3.93	1.10	4.58	0.77	4.04
CBGA	2.11	0.46	3.07	0.43	0.99	0.41	0.54	0.32	23.50	0.50	3.25	0.41	4.91	0.45	3.26	0.42	2.24	0.43
CBG	0.13	0.16		0.28		0.12	0.11	0.82	0.26	2.57	0.14	0.58	0.17	0.93	0.05	0.48	0.14	0.85
CBN	0.44	1.13	0.35	0.11	0.38	0.13	0.04			0.14	0.08					0.33	0.07	0.08
Δ-9-THC	0.10	0.01	0.13	0.11	0.09	0.05	0.01				0.07	0.02	0.16		0.14	0.02	0.05	
CBC	0.46	0.05	0.34	0.27	0.25	0.15				0.56	0.13	0.65				0.01		
Δ-9-THCA	0.88	0.68	0.31	0.14	1.30	0.02	0.23	0.16	0.01	0.01	0.10	0.13				0.38		

**Table 10:** Distribution pattern of the glandular and non-glandular trichomes in the examined *C. sativa* varieties.

Plant Part	Trichome Morphotype	24 K	Gorilla Glue	Lemon Conti Kush	Fresh Mountain	Amnesia Cookies	Pablito	White Shark	Venon OG	Lemon Conti Kush New
**bract**	hair-like lithocysts	++	++	+	++	++	+	++	++	+
	bulbous	++	++	++	++	++	++	++	++	++
	capitate-stalked	++	++	++	++	++	++	++	++	++
	capitate sessile	+	+	+	+	+	+	+	+	+
**bracteole**	hair-like lithocysts	++	++	+	++	++	+	++	+	+
	bulbous	+	+	+	+	++	+	+	+	+
	capitate-stalked	++	++	++	++	++	++	++	++	++
	capitate sessile	++	+	++	+	++	++	+	++	++
**inflorescence axis**	hair-like lithocysts	+	+	+	++	++	++	+	+	+
	bulbous	+	+	+	++	++	++	+	++	+
	capitate-stalked	+	+	++	++	++	++	+	++	++
	capitate sessile	+	+	+	+	+	+	+	+	+

Trichome distribution: (+) present in all the replicates; (++) abundant in all the replicates.

## Data Availability

Not applicable.
